# The European Health Union: European Union’s Concern about Health for All. Concepts, Definition, and Scenarios

**DOI:** 10.3390/healthcare9121741

**Published:** 2021-12-17

**Authors:** Marie Nabbe, Helmut Brand

**Affiliations:** 1 European Hospital and Healthcare Federation (HOPE), Avenue Marnix 30, 1000 Brussels, Belgium; 2 Department of International Health, Care and Public Health Research Institute (CAPHRI), Faculty of Health, Medicine and Life Sciences, Maastricht University, 6229 ER Maastricht, The Netherlands; helmut.brand@maastrichtuniversity.nl

**Keywords:** European Union, public health, health mandate, European Health Union, scenario planning

## Abstract

The COVID-19 pandemic brought visibility and intensified the discussions on the European Union’s (EU) health mandate. The proposals of the European Commission (EC) to move towards a European Health Union (EHU) can be seen as a starting point towards more integration in health. However, the definition of what the EHU will look like is not clear. This paper searches to find a common definition, and/or features for this EHU through a systematic literature review performed in May 2021. “European Union’s concern about health for all” is suggested as a definition. The main drivers identified to develop an EHU are: surveillance and monitoring, crisis preparedness, funding, political will, vision of public health expenditures, population’s awareness and interest, and global health. Based on these findings, five scenarios were developed: making a full move towards supranational action; improving efficiency in the actual framework; more coordination but no real change; in a full intergovernmentalism direction; and fragmentation of the EU. The scenarios show that the development of a EHU is possible inside the current legal framework. However, it will rely on increased coordination and has a focus on cross-border health threats. Any development will be strongly linked to political choices from Member States.

## 1. Introduction

The idea of a “European Health Community”—or “White Pool” was raised in 1952 but went down with the European Community of Defense. The failure of this project illustrates the importance of context and political will in European integration [1]. The COVID-19 pandemic, as an international health emergency, brought a change in the international and European contexts. On 11 March 2020, the World Health Organization (WHO) classified COVID-19 as a global pandemic, the first due to a coronavirus [2]. As of 13 March 2020, Europe became the epicenter of the pandemic [3]. The pandemic highlighted existing problems in the European Union (EU) in the matter of health policies as inequities between and within Member States (MS), lack of preparation, or shortages of medicines. Moreover, preventive measures were uncoordinated and divergences appeared between MS [4]. This situation brought into light questions on the EU competencies in (public) health as currently defined by the Treaty of the European Union (TEU) [5] and the Treaty on the Functioning of the European Union (TFEU) [6]. 

The Article 168 of the TFEU states that “a high level of human health protection shall be ensured in the definition and implementation of all Union policies and activities” [6]. This statement is also present in the Article 35 of the Charter of Fundamental Rights of the European Union (CFR) [7]. Besides this statement, the EU does not have direct authority on health matters, which are less integrated than policies as the European Energy Union or the European Green Deal. Indeed, health competence is a prerogative of the MS and not a European primary competence, following the application of the principle of subsidiarity [8].

However, the EU is called to work in cooperation with MS regarding health matters and has to support them. It also shares competence with them regarding the topic of “public health” as stated in the Article 168 of the TFEU [6,9]. Moreover, the institutions rely on other legislation in order to provide a “high level on human health protection” such as the internal market as seen with the Tobacco Products Directive (2014/40/EU) [10]. The EU also approaches health through the principle of “health in all policies” (HiAP), defined as “the recognition that a broader range of factors, other than those traditionally addressed within the ‘health’ field, affect population health” [11]. 

The EU capacities of actions regarding health were developed in recent years [12]. The creation of the European Medicines Agency (EMA) (1995) and of the European Centre for Disease Prevention and Control (ECDC) (2005) are illustrations of the development of the topic on the EU stage. Those advances were pushed through previous health crises such as the swine flu (H1N1) (2009) or the severe acute respiratory syndrome (SARS) (2003). These crises and evolutions of the EU were also windows of opportunities for research to question the EU health mandate and its future [13,14,15]. 

Indeed, research on the possible developments of the European health mandate or on a healthcare union is not a new trend [16,17,18]. The novelty is the introduction of the term “European Health Union” in the spring 2020 after the realization of the weakness and fragmentation of EU powers in health during the COVID-19 pandemic [19]. Although the initial questions considered the EU response and its role during the pandemic [4,20], the discussion soon broadened to which actions should be undertaken at the EU level. The topic also became political with, for example, the call of the European Parliament (EP) for the “European institutions and the Member States to draw the right lessons from the COVID-19 crisis and engage in far stronger cooperation in the area of health” and for “a number of measures to create a European Health Union” [21].

The COVID-19 pandemic seems to have redivided the cards between what exists and what is wanted or needed in terms of health competence in the EU. It also challenged the vision of European citizens on the EU, bringing the realization that there is no real health competence at the EU level [9,22]. Recent opinion surveys show a will from European citizens to develop a European health policy [23]. The President of the European Commission (EC)—Ursula von der Leyen—followed this will by introducing the term EHU in her State of the Union address of 2020 [24]. This was the first political use of the term. After the address, the EC published the Communication “building a European Health Union: reinforcing the EU’s resilience for cross-border health threats” [25]. Three proposals to pave the road towards the EHU followed. The first one considers a regulation on cross-border health threats [26], the second aims at strengthening the ECDC [27] and the third reflects on a reinforced role for EMA in crisis preparedness and management for medicinal products and medical devices [28]. These proposals are still under discussion and can serve as a base for scenario-planning. 

Scenario-planning has been applied before by the European institutions as illustrated by the White Paper on the Future of Europe which entails five scenarios on the possible evolution of the EU [29]. The scenario-planning method is particularly relevant for topics with high uncertainty. To move forward on an idea, the stakeholders and policymakers need to have a common comprehension of the meaning of a EHU for the MS, the EU, and European citizens. Neiner et al. applied this method to public health and outlined four steps to create scenarios in public health:

1. Refine the sense of purpose
2. Understand the driving forces or key patterns and trends
3. Develop scenario plots
4. Plot strategy, rehearse, and converse [30].

As indicated by the authors, a scenario-planning does not have the purpose to predict the future, but to foresight possible foundations to start policy discussions and public debate [30]. Building up on this framework, this research aims to contribute to the debate on a EHU by studying how it can be defined and the possible paths towards its achievement. 

## 2. Materials and Methods

This study analyses the themes and arguments of the content of text documents and is not based on numerical data, it can thus be classified as qualitative. The first aim of this research is to identify the possible meaning(s) of the EHU, based on the Communication of the EC on “building a European Health Union: reinforcing the EU’s resilience for cross-border health threats”. As a second step, predetermined and unpredictable factors need to be identified. For this purpose, a literature review was performed following the Preferred Reporting Items for Systematic Reviews and Meta-Analyses (PRISMA) guidelines [31].

The expression “European Health Union” is relatively new. In order to find the most recent papers tackling its definition(s), this quotation was kept as a ‘stand-alone’ for the literature review. The quotation was applied in the online databases Web of Science and Google Scholar to identify the current key discussions on the topic. The search led to 122 hits in total, 11 from Web of Science and 112 from Google Scholar. In addition to the databases, other sources were added from the websites of the European Health Union [32], the European Commission European Health Union [33], and the European Parliament Research Service (EPRS) [34]. 

The databases were screened by one reviewer lastly on 28 May 2021 for data collection. The articles considered relevant on title were extracted and deduplicated. Documents were then excluded on different criteria: the full text was not accessible; the document was not written in English or was published before 2020. To be included, the articles needed to focus on the EU level as it is the scope of the EHU. They also had to discuss or define the EHU, the health mandate of the EU or the role of a specific EU institution or mechanism in the health competence. The articles dealing with the COVID-19 consequences outside the health competence or the EHU were excluded. 

**Figure 1:**
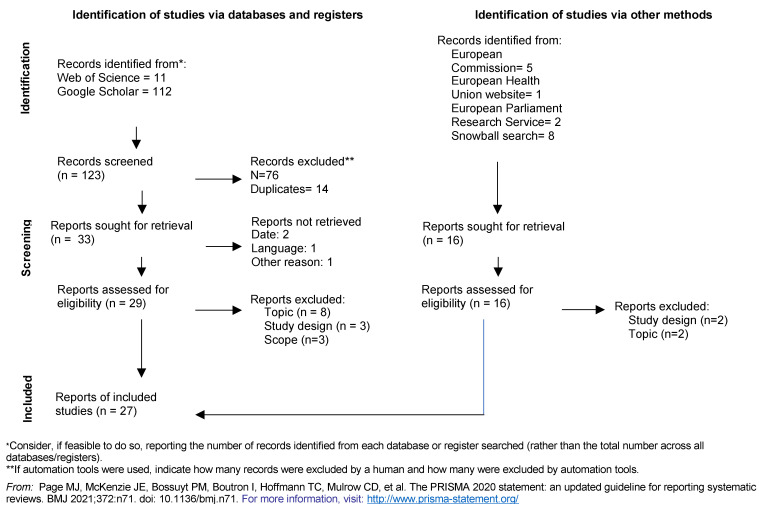
Prisma flow chart Reprinted with permission from ref. [38]. Copyright 2020 copyright PRISMA.

After the full-text eligibility, the bibliographies of the selected documents were screened on title to identify potentially missing articles in a snowball process. The articles retrieved from this process were screened for full-text eligibility as well. The process is illustrated in the PRISMA flow chart diagram in the results section (Figure 1). 

The data extraction was summarized in a table presenting (1) the title, (2) the authors, (3) the journal and the year of the article, and (4) the concepts identified. A critical appraisal was conducted based on the JBI checklist for text and opinion papers [35] or through the Scale for the Assessment of Narrative Review Articles (SANRA) [36]. No critical appraisal was conducted for legal or policy documents. 

After the identification of concepts and components of the EHU, the data analysis consisted in labelling several predetermined and unpredictable factors (the drivers), which will play a role in the elaboration of the scenarios. According to Neiner et al., “predetermined forces are the driving forces that we are relatively sure of and that we can predict” [30]. Once the previous steps are achieved, alternative scenarios can be developed considering the drivers identified in two tables following the model presented in Table 1.

The validity of this study is ensured by the application of the criteria of a scenario-analysis including plausibility, consistency, comprehensibility and traceability [37]. One of the core aspects of the scenario-planning is the unpredictability which affects the reliability of the work. The different biases that can affect the results will be identified in the discussion. 

## 3. Results

### 3.1. Systematic Literature Review

After full-text eligibility, 15 articles were included from the databases’ search and 12 from other methods. They were then reported in the PRISMA flow chart (Figure 1). Articles were excluded on topic (*n* = 10), on study design (*n* = 5) and on scope (*n* = 3). 

### 3.1.1. Components and Concepts of a European Health Union

Two main categories were identified in the literature to define the EHU: the components it can entail and the possible paths towards its achievement. 

The start taken by many articles was to define the scope of the health policy competence of the EU nowadays [26,39,40,41,42]. The current EU agencies, the ECDC and EMA should be reinforced [25,27,28,43]. A new agency or executive coordinating structure should be created—the European Health Emergency Preparedness and Response Authority (HERA) [39,44,45]. According to the literature, new threats are to come and better preparation is necessary [46,47,48,49]. As a consequence, the EU requires better pandemic preparedness, risk assessment and surveillance as well as data sharing [44,45,47,50,51,52]. Better risk management is also needed [43,45,46,47,51]. Moreover, the EHU should be included in a wider public health approach. It is linked to movements or concepts such as One Health [44,46,53], Global Health [39,54,55], Sustainable development goals (SDGs) [39,54] or Determinants of health [54,56]. There is a conception of public health, and health security, as a public good and the EU is seen as the appropriate level to provide it [40]. This vision is linked to the SDG3 and can relate to the promotion of well-being as a demand for a future EHU [39,53]. 

To achieve the EHU, there are two major key points. The first one is the political will as no policy can be achieved without it [12,40,51,53,54,57,58]. The second is the funding [40,41,43,44,45,50,51,55,59]. The EU4Health program, which is now independent from the European Social Fund, illustrated the strong negotiations that can take place regarding funding with an important difference between what was proposed by the EC and the response of the European Council [40,56]. Moreover, the current legislation should be better used and the EU governance system stronger [44,53]. The EU4Health Policy framework reminds that “a high level of health human protection is to be ensured in the definition and implementation of all Union policies and activities”, making health a question for the whole European governance as a whole following the application of the HiAP principle [53]. Innovative solutions could be applied as defining public health as a cross-border problem [12,42,48,49]. Institutionally, innovation is needed as well [52]. Regarding crisis response, binding coordination or mechanisms for MS could be developed to ensure the EU’s coordination [12,51]. However, a full centralized approach is not considered necessary [52]. This could be counteracted by the use of intergovernmental mechanisms as the Joint procurement (JPA) [43,58]. Lastly, a treaty change is envisioned by some authors with the possibility to insert the EHU in the Treaty’s text [39,46].

### 3.1.2. The Definition of a European Health Union 

Although all publications’ analyses describe or relate to components of the EHU, none try to give a definition encompassing the scope of the concept. The main documents giving wishes of its form are the policy documents of the EU or the Manifesto for a European Health Union. However, by taking these documents and the components mentioned previously into account, a central frame can be seen. This results in the suggested definition of the EHU as “European Union’s concern about health for all”, based on a definition of Public Health that has been elaborated on earlier [60]. Indeed, the EU4Health Programme, the Article 168 of the TFEU or the Article 35 of the CFR all mentioned the following statement: “a high level of human health protection shall be ensured in the definition and implementation of all Union policies and activities”. As the EU’s (health) policies are more and more interlinked with global (health) issues, it is not only any more about the health of the EU citizens. The COVID-19 pandemic has made it clear: if Europe does not care about the immunization progress in other continents, virus variants will develop there and will be re-imported to Europe. 

## 3.2. Driving Forces, Key Patterns, and Trends 

The creation of a EHU will be influenced by several predetermined forces. Firstly, the EU already has a role in surveillance and monitoring through European agencies as the ECDC. However, the agency’s capacities are undermined by a lack of funding and personnel [44,51]. Its reinforcement, envisioned by the EC, would require more funding and possibilities of action to coordinate the MSs’ actions [27,43]. The strengthening of national surveillance is also an important action to undertake [43,52]. At the beginning of the COVID-19 pandemic, the ECDC failed to detect the seriousness of the threat and the lack of preparation of the MS. This failure was linked to a lack of data sharing and reporting from the MS [50,51], and aligns with the importance of crisis preparedness. Supporting the MS in crisis management is already a EU’s role [52]. However, the recommendations made by the EU are non-binding. This resulted in a lack of coordination in the MSs’ public health actions and medical countermeasures during the COVID-19 pandemic [51]. Several actions could have been, and should be, taken before a pandemic occurs. Some possibilities are the revision and better supervision of national preparedness plans, the development of stronger cooperation and the focus on coordination with for example the reinforcement of the Health Security Committee (HSC) [47,51,57]. The envisioned HERA would work on improving crisis preparedness and coordination with the other agencies [45]. To ensure the work efficiency of the European agencies and of a EHU, the funding is a major issue where different mechanisms can play a role. First, the new EU4Health Programme is identified as a “fundamental shift in EU’s approach to health systems” [50]. Although this program is the largest health program to date, and is set to be independent [40], the financial situation of the agencies as the ECDC should have a sound base and not depend on project funding. The JPA could also be expanded and be a step forward in the development of the EHU [58]. As a voluntary procedure, it would focus on a more intergovernmental side of a EHU development. Lastly, State Aid laws could potentially be used for a EHU, either through MS or with a EU contribution [59]. 

Some unpredictable factors can also be identified, although the list is not exhaustive. The first element is the need of political will from the EC and the MS to increase the EU health action. The legal basis in the EU is described as sufficiently important to develop the EU’s actions in health [12]. However, a full political will is currently lacking which makes a treaty change difficult to envision [42]. Political choices and outcomes of political debates on the topic will play an important part in the development and/or direction of a EHU [19]. Linked to policies and politics, the vision of public health expenditures by national politicians and governments is also important to envision the development of a EHU. Since the financial crisis of 2008, public health is rather seen as a cost than an investment [44]. A decline was observed in health expenditures as well as in preventive care [56]. This led to important cuts in the healthcare sector, reduction of investment in research as well as in preparedness strategies [44,59]. The readiness of public actors to invest in public health affects the development of a EHU and the preparedness to future (health) threats. The population awareness and interest in the topic may challenge this vision. The first phases of the COVID-19 pandemic displayed a “widespread public criticism of the Union for apparently failing to support its own Member States” [12]. There is now an increasing recognition that the EU does not have the primary competence in health. This topic is a growing concern among European citizens [40,44]. The concerns and wishes of the European population are measured through the Eurobarometers for example. The Eurobarometer 94 of winter 2020–2021 showed that “close to four EU citizens in ten consider health as the most important issue facing the EU” [23]. Since its introduction in the Eurobarometer in summer 2020, health has known an increase of 16 percentage points and is now mentioned by 38% of respondents [23]. The EU can be influenced by the bottom, its citizens, but also by the top with international action and global health. The implementation of the International Health Regulations (IHR) was an issue during the COVID-19 pandemic [44,56]. Moreover, the EU has a responsibility towards global health and international cooperation [39,44]. A EHU could strengthen the role of the EU on the global stage and the interconnections of health with other policies could be used to set international standards [54].

## 3.3. Scenario Plots 

Five scenarios were developed from the previously identified drivers. They were chosen through a discussion in a focus group and were kept similar to the previous scenarios identified at the EU level as in the White Paper on the Future of Europe [29]. The key element of the scenarios is the level of involvement from the MS. If the commitments of the EP and the EC are important, the MS willingness of action will be decisive. The first and fifth scenarios represent the edges of the possible spectrum, as they imply major political and legal changes. Indeed, Scenario 1 would be in the direction towards a federal Union, while Scenario 5 would be the disappearance of the EU as we know it. The second, third and fourth scenarios are based on different directions that could be chosen by the MS and the European institutions. They go from a supranational power development towards a more intergovernmental framework. While going in different directions, these three middle scenarios imply a coordination between the MS. The major differences are the amount of power they are ready, or not, to delegate. The different scenarios are presented in Table 2 and Table 3. The tables present the drivers on the left side and display the different possibilities of evolution of these factors through each scenario lens. 

## 4. Discussion

The suggested definition for a EHU is “European Union’s concern about health for all”, close to the concept of “health for all” of the WHO [61]. The mandate of the EU in public health grows regularly since the 2000s [51]. However, the EU is a large machine that moves forward slowly. Major health policy integration shifts in the EU happened after crises [53]. The ECDC was for example created in 2005, following the uncoordinated and inefficient response of the EU to the SARS in 2003 [40]. This mechanism of ‘failing forward’ and building policies around a crisis is regular at the EU level [62,63]. The COVID-19 pandemic is a crisis that highlighted the limits of the EU system in public health and crisis management. It might become “a game-changer on the acceptation of health in European policy” [19,47]. However, it still follows the ‘failing forward’ mechanism.

If the pandemic is the trigger, it seems logical that the opening part of the discussion on the EHU is the response to the COVID-19 crisis and, in extension, to cross-border health threats. This is illustrated by the Communication and the three proposals of the EC published on 11 November 2020, which are direct responses to the current threat [25,26,27,28]. Although crisis management and cross-border threats seem to be the first part of a EHU, the use of the narrative is important. The use of the expression “European Health Union” to signify an expansion of the EU health mandate suggests a more integrated approach in health, with a stronger supranational power. The recent discussions about prevention, promotion, health security and global health made clear that the EU and its MS cannot guarantee health for all for their citizens on their own. They need to consider the wider determinants of health, neighboring countries, and a global approach. The actual competence and tool to be used is the HiAP approach which is already included in the treaties. However, this approach has its limits. The Manifesto for a European Health Union builds on the discussion on the strengthening of the EU power, taking inspiration from the CFR and the EU pillar of Social rights [46]. Going much further than the EC proposals, it raises the question on how much more the EHU should entail to achieve health for all in the EU [57].

The first scenario follows this idea and presents an option going further than the proposals of the EC. It encompasses a more integrated approach to health and goes beyond cross-border threats and crisis management. To realize this EHU, a full-scale treaty change is required, which seems unlikely [40]. This would indeed require a full consensus between MS as it is a unanimity vote. Moreover, the support of the EU citizens would not be guaranteed in a climate of Euroscepticism. The realization of this scenario at short or medium range is highly unlikely. However, the current legal base of the EU already provides possibilities to develop a stronger health-focused Union and more coordination [12,40,41]. This idea is the core of the second scenario, showing the range of possibilities if there is enough political will. Vervoort and Van Daalen introduced the idea of seeing public health itself as cross-border threat, rather than a component of health systems [48]. This perspective changes the focus without modifications of the legal basis, which is dynamic enough to create a EHU [64]. The third scenario echoes to past situations in the EU. Although agencies were created, lessons were not sufficiently drawn from previous crises. A new crisis could be a game-changer for this scenario as it could change the political focus towards another domain. Public health would be back in its box until the next pandemic or public health challenge. The fourth scenario does not automatically imply the oblivion of public health, but more the decision of national governments to use intergovernmental mechanisms or inter-national coordination tools to act on public health issues. The development of the HSC or the reinforcement of neighboring agreements could be examples of applications for this scenario. However, coordination in an intergovernmental framework remains limited by nature. It does not seem up to the new challenges faced by the EU in providing joint and timely responses to large scale-up pandemics [51]. Lastly, the fifth scenario encompasses the Brexit example, where national governments decide to leave or shut down the EU level. Although this possibility is to keep in mind for the debate, the realization of this scenario appears unlikely with the increase of MS cooperation and coordination during the COVID-19 pandemic.

The EC’s response to the COVID-19 pandemic—reinforcing the powers of the current agencies and creating a new agency, HERA—follows the previous patterns of creating a new agency for a new crisis [45]. The new agency was confirmed by the EC’s decision on 16 September 2021 [65]. However, if the COVID-19 pandemic showed something, it was that all problems were not resolved with the creation of agencies. Although the COVID-19 pandemic as it is was an unpredictable factor, the threat of an epidemic was warned by experts [44]. The agencies gave support during the crisis, but lessons were not learned from previous crises. The EU and its MS need to learn from their mistakes, but political will is necessary to implement changes. The new EU4Health Program (2021–2027) is an illustration of the divergences of will and of the uncertainty of a EHU direction [66]. While presented as a milestone with the highest budget to date for a health program, the difference of funding between the proposition of the EC (€9.4 billion) and the response from the Council (€1.7 billion) is striking in the core of the COVID-19 pandemic. Although some actions towards a EHU are presented as uncontroversial by the literature, such as the reinforcement of the ECDC or of the HSC [40], the realization and the efficiency of all health policy action need to be taken cautiously. This is reinforced by the absence of a common definition of “health” or “public health” at the European level. At the international level, the WHO definition of health was criticized and discussed, but it has the advantage to exist. This absence at the EU level brings more complexity. However, it also can be an opportunity to define it all together and to debate on what European citizens, stakeholders, and politicians want. This will to open the debate at all levels of the EU is illustrated through the Conference on the Future of Europe [67]. The digital platform launched on 19 April 2021 shows health as one of the ten topics discussed [68]. The EC is supposed to reach conclusions from the conference regarding the future of Europe in spring 2022.

This research has some limitations. First, the scenario-planning method itself implies a certain degree of confusion bias as it is a subjective creation. The risk of bias was however limited by the literature review. The review includes a relatively high number of opinion papers that carry the subjectivity of the author(s). To increase the reliability of this scenario-planning, stakeholders’ consultations could be carried upon which was not possible in the scope of a master’s thesis. This leads to recommendation for further research. This scenario-planning is thought as an introduction to the topic. The next step of the research would be to conduct interviews with stakeholders and experts in the field. A RAND/UCLA Appropriateness Method could be performed [69]. Otherwise, a Delphi round, as recently realized on the “scientific, technological and socio-economic conditions of the end of the COVID-19 crisis” by the EC Directorate-General for Research and innovation [70], could be applied to give more depth to the scenarios.

## 5. Conclusions

The previous advances in EU health competence have been developed after crises. As a direct reaction to the COVID-19 pandemic, the EHU as envisioned by the EC follows this pattern and has a strong focus on cross-border health threats. However, the suggested definition of the EHU has “EU’s concern for health for all” can go beyond cross-border threats and crisis management. The scenarios developed in this study show that, following the drivers, different paths are possible to achieve a EHU. In the coming years, a treaty change does not seem realistic but the development of a EHU is possible inside the current treaties, depending heavily on political choices and climate. The main issue is to find common ground on what is wanted regarding health at the EU level. In this sense, debates on the topic and exchange on the willingness of stakeholders, EU institutions, MS and European citizens for the future should be encouraged to move forward on the EU health competence. 

## Figures and Tables

**Table 1:** Scenario template.

Factors/Drivers	Example: Making a Full Move towards Supranational Action	Scenario 1	Scenario 2	Scenario 3	Scenario 4
Example of pre-determined factor: funding	Funding is thought to support fully the supranational level.				
Pre-determined factor 1					
Pre-determined factor 2					
Unpredictable factor 1					
Unpredictable factor 2					

**Table 2:** Scenario planning for the development of a European Health Union.

		Scenario 1 Making a Full Move towards Supranational Action	Scenario 2 Improving Efficiency in the Actual Framework	Scenario 3 More Coordination but No Real Change	Scenario 4 in a Full Inter-governmentalism Direction	Scenario 5 Fragmentation of the European Union (EU)
Predetermined forces	Surveillance and monitoring	The European Center of Disease Control (ECDC) has the power to coordinate the action of all Member States (MS).	The MS give regular and up-to-date reports to the ECDC and coordinate their actions following the agency recommendations. Binding possibilities.	Merely incentives to encourage MS to deliver data. ECDC support.	The MS coordinate on their own or through intergovernmental mechanisms.	Coordination is at its lowest, and surveillance and monitoring are managed only at the national level.
	Crisis preparedness	A new agency (e.g., European Health Emergency Preparedness and Response Authority (HERA)) is at the center and coordinate MS and EU actions.	Having binding coordination plans but leaving the decision-making to the MS. Possible extension of the Health Security Committee (HSC) and creation of HERA.	Staying on incentives.	Crisis preparedness at the national level. No EU coordination plans. Possibility of coordination between neighboring countries.	Crisis preparedness at the national level. Strictly bilateral agreements.
	Funding	Funding is thought to support fully the supranational level.	Funding is made sufficient to support the action of the European agencies and European research to its best.	Funding is insufficient to support the planned European actions. The level of funding is non-consensual between the European institutions and/or the MS.	Funding of the EU level is kept at a minimal level and stays at MS level.	Funding is invested back at national level.

**Table 3:** Scenario planning for the development of a European Health Union.

		Scenario 1 Making a Full Move towards Supranational Action	Scenario 2 Improving Efficiency in the Actual Framework	Scenario 3 More Coordination but No Real Change	Scenario 4 in a Full Intergovernmentalism Direction	Scenario 5 Fragmentation of the European Union
Unpredictable forces	Political will	The MS all agree to develop EU action in public health. The President of the European Commission (EC) is ready to continue in the same direction and change the EU treaties to recognize the importance of health. The EC continues its engagement towards health.	The MS decide with the EC to develop the EU action in public health inside the current treaties provision and agree to follow the EC’s lead as long as the national competence is respected.	Divergences between MS and between the European institutions. Change of the importance of public health depending on the political agenda.	The MS decide to keep full public health power and action at the national level.	Euroscepticism is at its fullest and the European level is removed from the equation.
	Vision of public health expenditures	Public health is envisioned as an investment for protecting all EU citizens.	Vision of public health evolves towards investment.	Public health is still envisioned mainly as a cost at the European and national levels.	No willingness to invest at the European level.	No willingness to invest at the European level.
	Population interest and awareness	European citizens ask for more competence at the EU level and expect a European coordinated action. They are aware of the possibilities of European public health.	European citizens ask for more competence at the EU level and expect a European coordinated action. They are aware of the possibilities of European public health.	Differences between awareness and knowledge of European citizens on EU health competences.	Lack of knowledge of the EU competence and/or disinterest for the EU level of action.	Lack of knowledge of the EU competence and/or disinterest for the EU level of action and/or important Euroscepticism.
	Global health	The EU can speak and act as one voice because of the development of a central competence.	Possible use of other legislations to act on global health and set standards. Intend for more common statements between MS.	No real position of the EU on global health. Difficulty to coordinate with international agencies.	No European position through the EC or institutions. Possible coordination between some countries or through the World Health Organization (WHO).	No European position.

## Data Availability

Not applicable.

## Abbreviations

CFR	Charter of Fundamental Rights
EC	European Commission
ECDC	European Centre for Disease Prevention and Control
EHU	European Health Union
EMA	European Medicines Agency
EP	European Parliament
EPRS	European Parliament Research Service
EU	European Union
HERA	European Health Emergency Preparedness and Response Authority
HiAP	Health in All Policies
HSC	Health Security Committee
IHR	International Health Regulations
JPA	Joint Procurement Agreement
MS	Member States
PRISMA	Preferred Reporting Items for Systematic Reviews and Meta-analyses
SANRA	Scale for the Assessment of Narrative Review Articles
SARS	Severe Acute Respiratory Syndrome
SDGs	Sustainable Development Goals
TEU	Treaty of the European Union
TFEU	Treaty on the Functioning of the European Union
WHO	World Health Organization
